# Aspirin upregulates αB-Crystallin to protect the myocardium against heat stress in broiler chickens

**DOI:** 10.1038/srep37273

**Published:** 2016-11-18

**Authors:** Shu Tang, Bin Yin, Erbao Song, Hongbo Chen, Yanfen Cheng, Xiaohui Zhang, Endong Bao, Joerg Hartung

**Affiliations:** 1 College of Veterinary Medicine, Nanjing Agricultural University, Nanjing 210095, China; 2 Institute for Animal Hygiene, Animal Welfare and Farm Animal Behaviour, University of Veterinary Medicine Hannover, Foundation, Hannover, 30173, Germany

## Abstract

We established *in vivo* and *in vitro* models to investigate the role of αB-Crystallin (CryAB) and assess the ability of aspirin (ASA) to protect the myocardium during prolonged heat stress. Thirty-day-old chickens were divided into three groups (*n* = 90): heat stress (HS, 40±1 °C); ASA(−)HS(+), 1 mg/kg ASA orally 2 h before heat stress; and ASA(+)HS(−), pretreated with aspirin, no heat stress (25 °C). Hearts were excised after 0, 1, 2, 3, 5, 7, 10, 15 and 24 h. Heat stress increased body temperature, though the ASA(−)HS(+) group had significantly higher temperatures than the ASA(+)HS(+) group at all time points. Compared to ASA(+)HS(+), the ASA(−)HS(+) group displayed increased sensitivity to heat stress. Pathological analysis revealed the ASA (+)HS(+) myocardium showed less severe changes (narrowed, chaotic fibers; fewer necrotic cells) than the ASA(−)HS(+) group (bleeding and extensive cell death). *In vitro*, ASA-pretreatment significantly increased primary chicken myocardial cell survival during heat stress. ELISAs indicated ASA induced CryAB *in vivo* to protect against heat stress-induced myocardial damage, but ASA did not induce CryAB in primary chicken myocardial cells. The mechanisms by which ASA induces the expression of CryAB *in vivo* and protects the myocardium during heat stress merit further research.

Heat stress is an environmental and occupational hazard. The prevention of deaths caused by extreme high temperatures (above 42 °C) is an issue of human public health importance and also a concern in animal production^1^. In 1995, a heat wave in Chicago resulted in 700 reported deaths, of which 39% occurred in individuals known to have a prior heart condition^2^. Similar reports indicated extreme temperatures caused a large number of deaths and heat-related injuries in Europe in 2003 and in California in 2006^3^,^4^. Mammals have sweat glands, but can still be affected by high temperatures and even suffer sudden death. The conception rates of lactating Brown Swiss, Jersey and Holstein cows in the United States were reported to decrease from 52% to 32% as the maximum air temperature increased from 23.9 °C to 32.2 °C during the summer^5^. In Brazil, the pregnancy rates of Holstein cows in free stall reduced from 71.2% in the winter to 45.7% in the summer^6^.

High temperatures can also harm poultry, especially broiler chickens, which have no sweat glands and poor thermotolerance^7^. Heat stress causes a series of physiological and metabolic changes in broiler chickens, including elevated body temperature, panting, respiratory alkalosis and sudden death^8^. According to our previous *in vivo* and *in vitro* research in rats, sudden death caused by heat stress (41 ± 1 °C) is primarily the result of pathological changes in the heart, such as necrosis and cell degeneration^9^. The heart is a critically important organ in animals and humans. Previous clinical reports demonstrated that thermal tolerance to heat stress is impaired in human patients with cardiovascular disease. In particular, patients with cardiovascular conditions associated with ventricular dysfunction and chronic heart failure (CHF) are predisposed to heat intolerance^10^,^11^. Despite the fact that high heat stress-induced mortality rates of nearly 40% have been reported among broiler chickens exposed to a temperature of 40 °C, little attention has been paid to investigation of the mechanisms associated with heat stress in broiler chickens.

The heat shock response (HSR) is an evolutionarily-conserved defense mechanism that protects against sudden stresses, such as elevated temperature or environmental changes^12^. Heat shock proteins (HSP) are a family of proteins that are produced by cells in response to exposure to stressful conditions. They were first described in relation to heat shock^13^, but are now known to also be expressed during other stresses including exposure to cold^14^, UV light^15^, and during wound healing or tissue remodeling^16^. Many members of this group perform chaperone function by stabilizing new proteins to ensure correct folding or by helping to refold proteins that were damaged by the cell stress^17^. HSPs are found in virtually all living organisms, from bacteria to humans. Heat-shock proteins are named according to their molecular weight. For example, Hsp60, Hsp70 and Hsp90 (the most widely studied HSPs) refer to families of heat shock proteins on the order of 60, 70, and 90 kilodaltons in size, respectively^18^. Some previously researches transfected Hsp90, Hsp60 and Hsp56 into rat H_9_C_2_ cells and cultured primary cardiac cells to investigate the mechanisms related to stress responses. Interestingly, these experiments clearly indicated that overexpression of Hsp90 protected against a number of stimuli^19^,^20^. Our other studies^21^ indicated overexpression of Hsp70 exerted a protective effect against heat stress^22^,^23^. Therefore, these studies indicate the individual Hsps exert specific protective effects and their functions should be assessed separately. However, the protective roles of Hsp60 and other Hsps have not been fully investigated^24^,^25^.

The small heat shock protein αB-Crystallin (CryAB, HSPB5, 20 KDa) belongs to the small heat shock protein (sHsp) family, and is expressed in most organisms in response to several types of stress (e.g., UV, hyperthermia, toxic radicals) in order to protect cells^26^,^27^. CryAB is expressed ubiquitously throughout the mammals’ body and exerts a variety of highly protective functions to maintain homeostasis. CryAB has also been shown to exert anti-apoptotic properties, as it can prevent cell death in response to conditions such as stroke^28^ by maintaining the cell cytoskeleton^29^. Mechanistically, CryAB localizes to the I-band and M-line region in myofibrils and has been confirmed to play a myofibril-stabilizing role in cardiomyocytes *in vitro*^30^. It is now believed that the pleotropic functions of CryAB are the result of its diverse interactions with a wide variety of different proteins^31^. In our previous research, we investigated CryAB expression and localization, and demonstrated it plays a protective role against heat stress in rat myocardial cells *in vivo* and *in vitro*^21^. However, the function of CryAB remains poorly explored compared to other members of the sHsp family.

Aspirin (acetylsalicylic acid, ASA) is widely used as a drug to treat pain, fever and inflammation^32^ and is also prescribed long-term at low doses to help prevent heart attacks, strokes and blood clot formation in high risk individuals^33^. Therefore, ASA is an important part of the care of patients who have suffered a myocardial infarction (heart attack)^34^. ASA has been reported to reduce the development, growth rate, or both, of several human cancers in animal models^35^, via a mechanism mediated at least in part by inhibition of the cyclo-oxygenase (COX) enzymes and reduced production of prostaglandins and other inflammatory mediators^36^. Interestingly, heat stress has been shown to active multiple cellular responses, including inflammatory pathways^35^.

In present study, we established *in vivo* and *in vitro* models to investigate the functions on the chicken myocardium during prolonged heat stress. Moreover, we also assessed the ability of ASA to protect the myocardium against heats stress *in vivo* and *in vitr*o, and investigated the relationship between CryAB and ASA during heat stress.

## Materials and Methods

### Establishment of the *in vivo* heat stress model

All experiments were performed in accordance with the guidelines of the Animal Ethics Committee of Jiangsu Province (China) and were approved by the Institutional Animal Care and Use Committee of Nanjing Agricultural University, China. The *in vivo* heat stress model was established as previously described^37^. Briefly, one-day-old specific pathogen free (SPF) chickens were purchased from Qian Yuan Hao Biotechnology Company, Nanjing, China. The entire population was vaccinated against Newcastle disease (ND) and infectious bursal disease (IBD) on day 7 and day 14, respectively. The birds were allowed to acclimate to their new housing and recover from environmental stress for 30 days. Then, 270 chickens were randomly divided into three groups, designated the HS group (heat stress), the ASA(−)HS(+) group (pretreated with aspirin before heat stress), and the ASA(+)HS(−) group (pretreated with aspirin, no heat stress), respectively, with 90 chickens in each group. The chickens were not provided water for the 12 h leading up to the experiment. The chickens in the ASA(+)HS(−) and ASA(+)HS(+) groups were administered aspirin orally at 1 mg/kg body weight (ASA powder >98% purity; Sigma, USA) 2 h before the heat stress phase of the experiment. During the heat stress phase, the chickens in ASA(+)HS(−) group were housed under normal conditions as a control group (i.e. not exposed to heat), while the chickens in the ASA(+)HS(+) and ASA(−)HS(+) groups were exposed to heat stress by rapidly, gently moving the animals from 25 ± 1 °C to an air chamber (GJ-1, Suzhou Fengshi Laboratory Animal Equipment Co. Ltd, China) preheated to 40 ± 1 °C at 60~70% humidity. The broilers were allowed free access to food and water *ad libitum* during heat stress exposure. At 0, 1, 2, 3, 5, 7, 10, 15 and 24 h, 10 chickens in each group were sacrificed humanely by decapitation. The body temperature of the broilers was measured via the rectum (3 cm inside) using a mercury thermometer within 2 min before slaughter. The hearts were excised and fixed in 10% formalin for pathological studies or frozen in liquid nitrogen for ELISA.

For pathological analysis, heart samples from the 10 chickens in each treatment group were fixed in 4% methanol, paraffin-embedded, cut into 4 μm-thick serial sections, stained with hematoxylin (3 min) and eosin (1 min), and examined by light microscopy (Imager A2, ZEISS, Germany).

### Isolation and culture of primary chicken myocardial cells

All experiments were performed in accordance with the guidelines of the Animal Ethics Committee of Jiangsu Province (China) and were approved by the Institutional Animal Care and Use Committee of Nanjing Agricultural University, China. Twelve-day-old specific pathogen free (SPF) embryonated eggs (Qian Yuan Hao Biotechnology Company, Nan Jing, China) were opened. The hearts of the embryos were removed in a bio-clean environment, cut into pieces (1 mm^3^), washed in pre-cooled PBS (4 °C) four times, the fibrous tissue was digested in 1 mg/mL collagenase type I (17100-017, Gibco, USA) at 4 °C for 14–16 h, then the digestion was terminated by addition of Dulbecco’s modified eagle medium (DMEM; 11995-065, Gibco) containing 20% fetal bovine serum (FBS; 16141079, Gibco). The digest was centrifuged at 1000 rpm for 10 min, the cells were resuspended in DMEM containing 20% FBS, 100 units/mL penicillin and 100 units/mL streptomycin. The cells were cultured in cell culture plates at 37 °C in a humidified atmosphere of 5% CO_2_ and 95% air, then the primmorph were transferred into new cell culture plates after 1 h and 0.1 mmol/L 5-Bromo-2′-deoxyuridine solution (BRDU, B16880, Sigma, Germany) was added the culture solution. Cells were cultivated for 48 h to let them adhere.

Immunofluorescence analysis was employed to confirm the cells were myocardial cells using the marker alpha actinin (α-actinin, ab11007, abcam, USA) which is specific to myocardial cells. The proportion of myocardial cells was assessed in five randomly chosen fields of view (data not shown) by fluorescence microscopy (Cx41-32rfl, Olympus, Japan).

### Assessment of cell viability *in vitro*

Four groups of primary chicken cells were established (HS: 2 h, ASA + HS: 2 h, HS: 24 h, ASA + HS: 24 h). Cells (1 × 10^6^) were seeded and cultured in 6 cm cell culture plates (Corning, USA), and used in experiments when the cell division was higher than 85%. After treatment with ASA and/or HS, the cells were digested with 0.25% trypsin for 10 min, resupsended in DMEM containing 20% FBS and 1% penicillin/streptomycin, then counted using the Trypan blue assay under an inverted light microscope. Each sample was counted three times.

### CryAB ELISA

The chicken heart samples were washed in ice-cold saline and homogenized on ice in 10 volumes of homogenization buffer [0.15 M NaCl, 20 mM Tris-HCl (pH 8.0), 1 mM ethylenediaminetetraacetic acid, 1 mM phenylmethylsulphonyl fluoride, 0.1 μM E-46, 0.08 μM aprotinin, 0.1 μM leupeptin, and 0.1% NP-40] using an Ultra-Turrax homogenizer (623003, Fluko Equipment Shanghai Co. Ltd, China) The homogenates were centrifuged at 12,000 g for 20 min at 4 °C to remove debris, and the supernatant was collected and stored at −20 °C for protein quantification.

Primary chicken myocardial cells were washed twice with PBS and lysed in M-PERH mammalian protein extraction reagent (28501, Thermo Scientific, Waltham, MA, USA) supplemented with Halt^TM^ protease inhibitor cocktail (78425, Thermo Scientific, Waltham, MA, USA) according to the manufacturer’s instructions, centrifuged at 14,000 g for 5 min at 4 °C and the supernatants were used as total protein extracts.

The protein concentrations of the samples were measured using a Micro-BCA™ protein assay kit (23235, Thermo Scientific, Waltham, MA, USA), then CryAB protein levels were quantified using a commercially available ELISA Kit (MBS2882479, MyBioSource, USA) according to the manufacturer’s instructions.

### Statistical analysis

Curve Expert 1.3 software was used to generate the standard curves for the ELISAs. Data were compared with the baseline level (0 h in the HS group) by one-way analysis of variance (ANOVA) followed by Fisher’s least significant difference (LSD) test using SPSS version 21 for Windows and Graphpad prism 6.0 software. *P *< 0.05 was considered significant; *P *< 0.01 was considered highly significant. Duncan’s multiple range test was used to analyze CryAB levels between groups at each time point. All raw data presented are expressed as the mean ± standard deviation (SD). All experiments were repeated three times.

## Results

### Clinical manifestation of heat stress in broiler chickens

The broilers behaved normally after being orally administered ASA in water 2 h before heat stress. After 1 h heat stress, the broilers in the ASA(−)HS(+) group displayed polypnea and became more sensitivity to heat compared to the ASA(+)HS(+) pretreated group. After 5 h of heat stress, the broilers in the ASA(−)HS(+) group were not as active, and the ASA(+)HS(+) treatment group displayed similar to their behavior at earlier time point. From 10 h to 24 h of heat stress, the ASA(+)HS(+) group showed relaxed behavior compared to ASA(−)HS(+) group.

**Figure 1:**
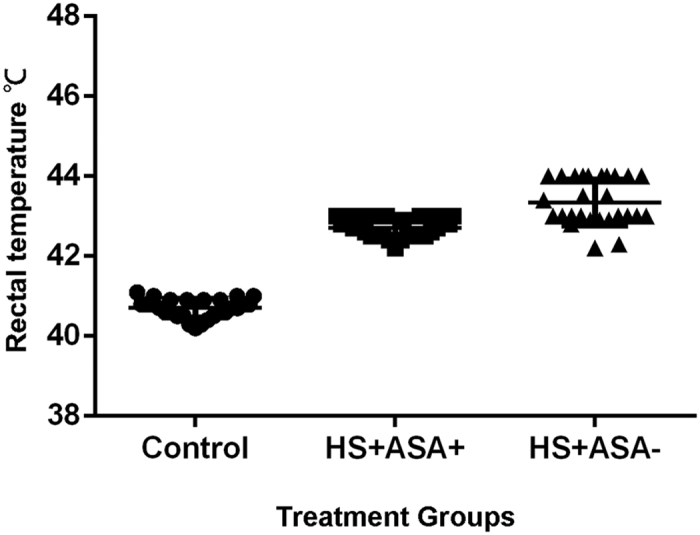
Rectal temperature of the chickens.

Both groups subjected to heat stress had higher rectal temperatures than the control group housed at RT (25 °C; Fig. 1); however, the ASA(−)HS(+) group had significantly higher body temperatures compared to the ASA(+)HS(+) group at all time points during heat stress (P* *< 0.05). The mercury thermometer used in this study ranged from 25 to 43 °C. During heat stress, the body temperature of most chickens in the ASA(−)HS(+) group was >43 °C (we assumed a body temperature of 44 °C).

### Histopathological changes in the myocardium in the *in vivo* model of heat stress

**Figure 2:**
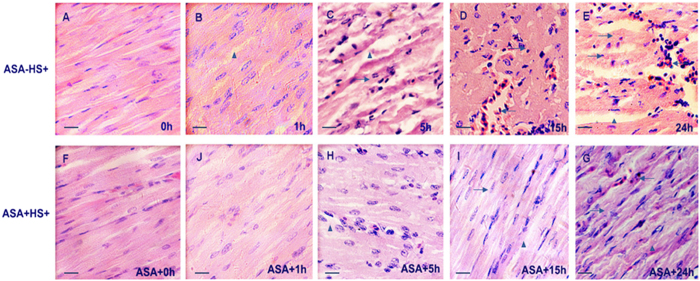
Pathological changes in the chicken myocardium after different durations of heat stress (1 bar = 10 μm). Chicken hearts were treated either by Heat stress or pre feed ASA (ASA+ Heat stress time). 1 h, 5 h, 15 h, 24 h treatment groups were stained with H&E and photographed using a Carl Zeiss optical microscope. (**A**) Control group (25 °C); (**F**) ASA pre treated control. As shown in (**A**,**F**), chicken myocardium showed no obvious pathological changes. These two groups showed no difference; (**B**) After 1 hour of heat stress, the space between cardiac fiber became wider and acute degeneration (▲) were observed in the cytoplasm compared to ASA(−)HS(+) control cells, the ASA(+) HS(+) group at 1 hour showed mild chaotic of cardiac fibers; (**C**) After 5 hours of heat stress, cell degeneration (▲) can be observed at nearly all views on the slide, and several cells showed karyopyknosis (→). In (**H**), the main pathological changes were cell degeneration (▲); (**D**) Karyopyknosis (→) accompanied by bleeding (←) can be abserved after 15 hours of heat stress. (**I**) Some necrotic (→) and cell degeneration (▲) were the main pathological changes after ASA pre treatment; (**E**) After 24 hours of heat stress, both ASA(−)HS(+) and ASA(+) HS(+) (**G**) showed bleeding and chaotic cardiac fibers, however in ASA(−)HS(+) group, more severe cell damage can be obviously observed. The ASA(+)HS(−) group was also observed in present experiment, there was no obvious pathological changes in all groups (data not shown).

The histopathological changes in the myocardium of the heat stressed groups and control group are shown in Fig. 2. In the present experiment, some chickens were pre-treated with ASA 2 h before heat stress, euthanized at 0 h of heat stress and defined as the ASA control group (Fig. 2F). As shown in Fig. 2C, severe damage to the heart tissue was evident after 5 h of heat stress. The space between muscle fibers became wider (▲) and karyopyknosis could be observed (→). In the ASA(+)HS(+) group, cell swelling was observed, accompanied by slightly wider spacing between the heart fibers. After 15 h heat stress (Fig. 2D), bleeding (←) and cell death were the major pathological changes. The heart tissue lost its normal organization and structure, with the cell cytoplasm fusing together, and nuclei disappearing or undergoing karyopyknosis. In the ASA(+)HS(+) group (Fig. 2I,G), the cardiac fibers became narrowed and chaotic after 15 h heat stress compared with the no heat stress control group, with fewer blood cells observed in the intercellular spaces. Most cells had degenerated, and a few were necrotic (→).

### Effect of heat stress on the survival of primary myocardial cells *in vitro*

**Figure 3:**
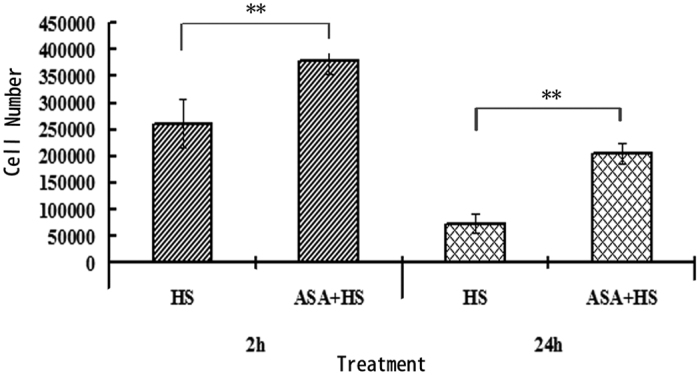


Primary chicken myocardial cells were treated with or without 1 mg/mL ASA ASA for 2 h, cultured at 42 °C for 2 h or 24 h, and cell numbers were determined (Fig. 3). After 2 h of heat stress, there was significantly more cells in the ASA pretreated group than the HS group (*P *< 0.01). After 24 h heat stress, the number of cells had reduced in both groups compared to the numbers after 2 h heat stress; however, significantly more cells had survived in the ASA pretreated group than the HS group after 24 h heat stress (*P *< 0.01).

### Effect of heat stress on CryAB protein expression *in vivo*

**Figure 4:**
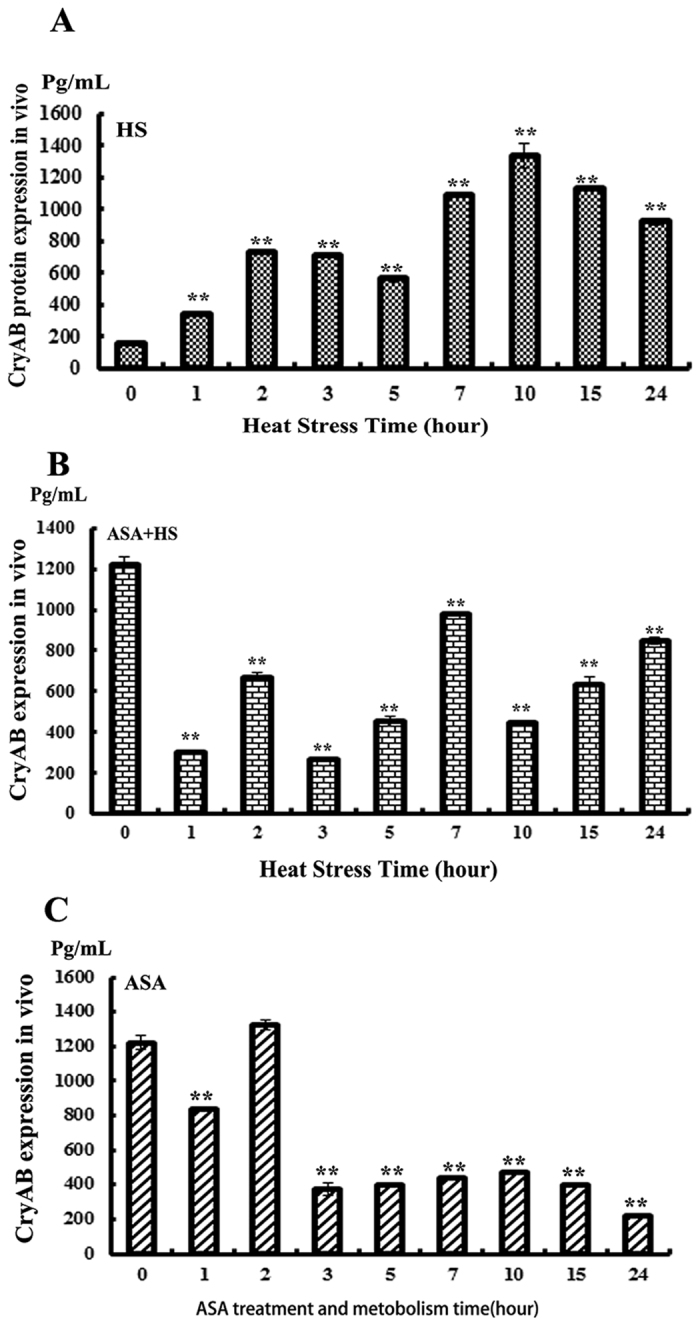
CryAB expression in the chicken myocardium during heat stress *in vivo.* (**P < 0.01).

The expression of CryAB was quantified in the myocardium of the chickens using an ELISA (Fig. 4). At 0 h heat stress, the concentration of CryAB in the myocardium of the control group was only 200 pg/mL compared to 1200 pg/mL in the groups pretreated with ASA for 2 h, which represents a 6-fold difference (*P *< 0.01).

In the HS group, expression of CryAB increased after 1 h heat stress, reached the highest level after 10 h of heat stress (1400 pg/mL), then slightly decreased but still remained high between 15 and 24 h heat stress (1000 pg/mL). In the ASA group pretreated with ASA for 2 h, CryAB peaked at 0 h and 3 h (i.e., 2 and 5 h after administration of ASA), then sharply decreased after 3 h (p* *< 0.01) and recovered to control levels (200 pg/mL) after 24 h heat stress. In the ASA(+)HS(+) group, the expression of CryAB followed a different trend. After 1 h of heat stress, CryAB was expressed at 6-fold lower levels compared to the HS group not pretreated with ASA, then slightly increased at 2 h, but remained lower than the levels in the ASA(−)HS (+) group up to the end of heat stress (24 h) with the exception of a peak at 7 hours (1000 pg/mL). At 3 h of heat stress, the level of CryAB was significantly lower in the ASA(+)HS(+) group than the HS group (p* *< 0.01).

### CryAB expression in primary chicken myocardial cells in the *in vitro* model of heat stress

**Figure 5:**
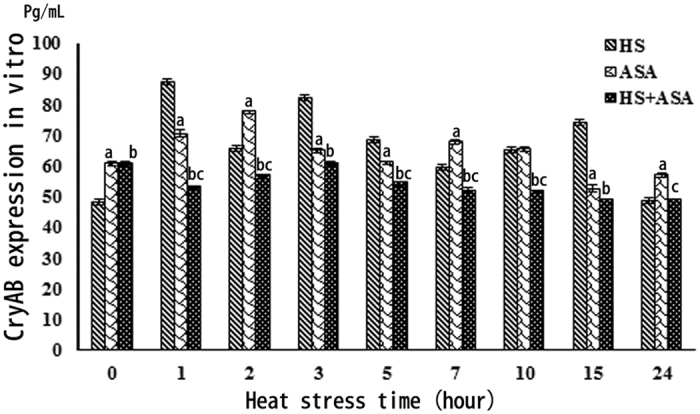
ELISA of CryAB expression *in vitro.* a: P < 0.01: ASA(+)HS(−) compared to HS group; b: P < 0.01: ASA(+)HS(+) compared to HS group; c: P < 0.01: ASA(+)HS(+) compared to ASA(−)HS(+) group.

CryAB expression was also measured in primary chicken myocardial cells *in vitro* after different treatments (Fig. 5). Before heat stress, CryAB was expressed at significantly higher levels in ASA-pretreated cells than control cells that had not been treated with ASA (p* *< 0.01). However, cells exposed to 1 h heat stress expressed significantly higher levels of CryAB compared to not only before HS (P* *< 0.01), but also compared to the ASA(+)HS(−) and ASA(+)HS(+) groups after 1 h heat stress. The levels of CryAB remained higher in cells subjected to heat stress [ASA(−)HS(+)] until 15 h compared to control cells. After 24 h heat stress, CryAB expression returned to the same level as control cells before heat stress. In the ASA(+)HS(+) group, CryAB expression decreased after 1 h and remained at a low level until 24 h heat stress.

## Discussion

Heat stress is a non-specific stressor that can affect the welfare of livestock and even contribute to death. Our previous research confirmed short term exposure to heat stress (1 h) induced detectable levels of enzymes related to heart damage, such as AST, CK and CKMB, in both the serum of rat blood and supernatant of rat myocardial cells^9^,^38^. Furthermore, pathological lesions, mainly due to necrosis, were observed in the rat myocardium after 40 min of heat stress *in vivo*, accompanied by lower CryAB expression, indicating CryAB may play an important role to protect the mammalian heart against heat stress^21^. In the present research, we investigated the expression profile of CryAB in the chicken myocardium using *in vivo* and *in vitro* models of heat stress lasting up to 24 h. Furthermore, we assessed the ability of ASA to protect the myocardium during heat stress, as it has been identified that the drug may protect the heart from heat stress by inducing Hsp90 and HspB1 expression^39^. The aim of this study was to determine whether CryAB could be induced by ASA and protect the myocardium during heat stress in broiler chickens using *in vivo* and *in vitro* models.

In the *in vivo* model, heat stress resulted in severe damage (observed after 5 to 24 h) in the myocardium; these changes were mainly characterized by karyolysis and necrosis, signifying cell death. In comparison, the myocardium was less severely damaged in the ASA pretreated group, in which degeneration of cytoplasm was observed after 1–15 h of heat stress and the myocardium became disorganized with a number of necrotic and karyolitic cells after 24 h. The body temperature of the chickens in the ASA(+)HS(−) group remained approximately 41 °C, which is the normal body temperature range for broilers. Heat stress increased the body temperature of the chickens; however, the ASA(+)HS(+) group maintained a lower body temperature (41–43 °C) than the ASA(−)HS(+) group (42–44 °C). ASA is widely used to treat pain, fever and cardiac disease in humans^36^. During heat stress, body temperature increases and homeostasis of the intestinal flora becomes disrupted, which may cause inflammatory factors such as TNF-α, IL-6, IL-8, among others, to be released into the circulation, which can lead to fever. ASA can involve into anti-inflammatory properties^35^. In the *in vivo* model, ASA played a key role to protect the heart from heat stress. Aspirin controls fever by acting on the prostaglandin system as it irreversibly inhibits the cyclooxygenase (COX) enzymes^36^. Pathological analysis confirmed pretreatment with ASA attenuated heat stress-induced damage to the myocardium *in vivo* and prevented heat stress-induced primary chicken myocardial cell death *in vitro.* The ability of ASA to protect the myocardium during heat stress is consistent with the fact that low dose ASA is widely used to reduce the risk of subsequent heart attacks and prevent the death of myocardial tissue^40^.

CryAB is a member of the small heat shock protein family (sHSPs, HspB1-HspB10) that is expressed in several organs and functions as a major cytoskeletal chaperone protein. The cytoskeleton plays a key role to maintain the physical shape of heart cells, protect against endoplasmic reticulum (ER) stress, and preserve mitochondrial function *in vivo* and *in vitro*. Mitochondria consume oxygen and provide ATP throughout the entire body, and occupy up to 60% of the volume of myocardial cells^41^,^42^. Mammalian CryAB expression and localization have been well-characterized both *in vivo* and *in vitro* in the rat^9^,^21^; these analyses indicated CryAB may play an important role in response to heat stress in cardiac cells. However, until now, the expression of CryAB had not been researched in poultry. Our *in vivo* expression profiling of broiler chickens revealed 2 h pretreatment with ASA increased the expression of CryAB in the myocardium by 6-fold compared to control chickens. CryAB reached the highest level after 2 h heat stress; however, CryAB expression decreased significantly (by nearly 4-fold) after 3 h heat stress in chickens pretreated with ASA. After 24 h heat stress, CryAB expression recovered to similar levels as untreated control animals (200 pg/mL) in the chickens pretreated with ASA. Exposure to heat stress in the absence of ASA resulted in the opposite trend: CryAB expression increased slightly after 1 h heat stress, reached the highest level after 10 h heat stress and remained high up to 24 h heat stress. These results suggest that pretreatment with ASA induced the expression of CryAB during the early stage of heat stress. This reflects the metabolism of ASA; the half-life of ASA in humans is approximately 2.0 to 4.5 h. In our *in vivo* model, the chickens were orally administered ASA 2 h before heat stress.

Pathological analysis of the *in vivo* model further confirmed that pretreatment with ASA to induce CryAB expression played a critical role to protect the myocardium against heat stress. In the ASA(+)HS(+) group, the expression of CryAB was strongly upregulated by pretreatment with ASA, slowly decreased during the initial stage of heat stress (1 h), slowly increased between 2 and 7 h heat stress, and then returned to similar levels as the HS group. CryAB has been shown to bind to other cytoskeletal proteins such as desmin and vimentin, which in turn can decrease CryAB expression^28^,^43^. The expression profiling suggested ASA only induced CryAB expression at the early stage (1–4 h) of heat stress.

The expression of CryAB was not the same *in vivo* and *in vitro* in primary chicken myocardial cells. CryAB did not dramatically increase at the beginning of heat stress (0–1 h) and did not sharply decrease after prolonged heat stress. Moreover, the heat stressed cells expressed higher levels of CryAB at several time points (1, 3, 5, 15 h) than ASA(+)HS(+) cells. In contrast to the *in vivo* model, ASA treatment induced lower levels of CryAB than HS *in vitro*. Unlike other Hsps (Hsp90, Hsp27) we have researched^39^,^44^, it may not possible to induce overexpression of CryAB *in vitro*. As previously stated, aspirin controls fever via the prostaglandin system by irreversibly inhibiting the cycloxygenases. However, cells in culture do not possess a hormone regulatory system. The main functional region of CryAB is its αB-crystallin chain, which means it cannot be induced by acid or other stressors like the inducible Hsps. However, CryAB could not be induced by pretreatment with ASA in primary chicken myocardial cells in the present study, which could reflect specific differences between the mammalian and chicken prostaglandin systems. ASA can induce Hsf1 expression, which binds to heat shock element (HSE) to induce the transcription of Hsp genes. However, unlike Hsp90, CryAB was not identified as a major Hsp that can be induced by Hsf1^45^,^46^. Similarly, CryAB showed no significant expression changes in primary chicken myocardial cells treated with ASA. Therefore, the mechanism by which ASA induces the expression of CryAB *in vivo* still needs to be identified. In conclusion, this study indicates that ASA can induce expression of CryAB and protect the myocardium *in vivo,* but not *in vitro*.

## Additional Information

**How to cite this article**: Tang, S. *et al*. Aspirin upregulates αB-Crystallin to protect the myocardium against heat stress in broiler chickens. *Sci. Rep.* **6**, 37273; doi: 10.1038/srep37273 (2016).

**Publisher’s note**: Springer Nature remains neutral with regard to jurisdictional claims in published maps and institutional affiliations.
